# Supplementary material for: Increased biosynthesis of acetyl-CoA in the yeast Saccharomyces cerevisiae by overexpression of a deregulated pantothenate kinase gene and engineering of the coenzyme A biosynthetic pathway
Source: Appl Microbiol Biotechnol. 2021 Sep 7;105(19):7321–37. doi: 10.1007/s00253-021-11523-4 (PMC8494682; doi:10.1007/s00253-021-11523-4)
Supplement: Supplementary file 1 — Supplementary file1 (PDF 131 KB) [file 253_2021_11523_MOESM1_ESM.pdf]

Applied Microbiology and Biotechnology

Supplementary Material

Increased biosynthesis of acetyl-CoA in the yeast *Saccharomyces cerevisiae* by overexpression of a deregulated pantothenate kinase gene

Judith Olzhausen[1], Mathias Grigat, Larissa Seifert[2],

Tom Ulbricht and Hans-Joachim Schüller

Center for Functional Genomics of Microbes,

Abteilung Molekulare Genetik und Infektionsbiologie,

Universität Greifswald,

Felix-Hausdorff-Strasse 8, D-17487 Greifswald, Germany

For correspondence:

Hans-Joachim Schüller, E-mail: schuell@uni-greifswald.de; Tel. +49 3834 4205703; Fax +49 3834 4205709.

Present address:

[1] Cendres+Métaux SA, CH-2501 Biel/Bienne, Switzerland

[2] Universitätsklinikum Hamburg-Eppendorf, Medizinische Klinik, Nephrologie

Supplementary Table S1: Complete list of *S. cerevisiae* strains.

Strain	Genotype
JS91.15-23	*MATα ura3 leu2 trp1 his3*
JS91.14-24	*MATα ura3 his3 cab1*$^{ts,\ G351S}$
JS19.1	*MATα ura3 leu2 trp1 his3 cab1Δ::HIS3* [pJO57: *ARSH4 CEN6 URA3 CAB1*]
LSY20	*MATα ura3 leu2 trp1 his3 cab4Δ::HIS3* [pGE7: *ARSH4 CEN6 URA3 CAB4*]
LSY21	*MATα ura3 leu2 trp1 his3 cab5Δ::HIS3* [pGE9: *ARSH4 CEN6 URA3 CAB5*]
BY4741	*MATa ura3 leu2 his3 met15*
MGY5<br>(derived from JS91.15-23)	*MATα ura3 leu2 trp1 his3* + pSH62 (2µm *HIS3 GAL1-cre*)<br>*HIP1::TPI1*$_{PRO}$-*HA*$_{3}$-*CAB2-CYC1*$_{TER}$
MGY12<br>(derived from MGY5)	*MATα ura3 leu2 trp1 his3* + pSH62 (2µm *HIS3 GAL1-cre*)<br>*HIP1::TPI1*$_{PRO}$-*HA*$_{3}$-*CAB2-CYC1*$_{TER}$<br>*ENO1::TPI1*$_{PRO}$-*HA*$_{3}$-*CAB3-CYC1*$_{TER}$
MGY14<br>(derived from MGY12)	*MATα ura3 leu2 trp1 his3* + pSH62 (2µm *HIS3 GAL1-cre*)<br>*HIP1::TPI1*$_{PRO}$-*HA*$_{3}$-*CAB2-CYC1*$_{TER}$<br>*ENO1::TPI1*$_{PRO}$-*HA*$_{3}$-*CAB3-CYC1*$_{TER}$<br>*MRH1::TPI1*$_{PRO}$-*HA*$_{3}$-*CAB4-CYC1*$_{TER}$
MGY16<br>(derived from MGY14)	*MATα ura3 leu2 trp1 his3* + pSH62 (2µm *HIS3 GAL1-cre*)<br>*HIP1::TPI1*$_{PRO}$-*HA*$_{3}$-*CAB2-CYC1*$_{TER}$<br>*ENO1::TPI1*$_{PRO}$-*HA*$_{3}$-*CAB3-CYC1*$_{TER}$<br>*MRH1::TPI1*$_{PRO}$-*HA*$_{3}$-*CAB4-CYC1*$_{TER}$<br>*ADH3::TPI1*$_{PRO}$-*HA*$_{3}$-*CAB5-CYC1*$_{TER}$
MGY18<br>(derived from MGY16)	*MATα ura3 leu2 trp1 his3* + pSH62 (2µm *HIS3 GAL1-cre*)<br>*HIP1::TPI1*$_{PRO}$-*HA*$_{3}$-*CAB2-CYC1*$_{TER}$<br>*ENO1::TPI1*$_{PRO}$-*HA*$_{3}$-*CAB3-CYC1*$_{TER}$<br>*MRH1::TPI1*$_{PRO}$-*HA*$_{3}$-*CAB4-CYC1*$_{TER}$<br>*ADH3::TPI1*$_{PRO}$-*HA*$_{3}$-*CAB5-CYC1*$_{TER}$<br>*ZPS1::TPI1*$_{PRO}$-*HA*$_{3}$-*CAB1*$_{W331R}$-*CYC1*$_{TER}$
MGY20<br>(derived from MGY16)	*MATα ura3 leu2 trp1 his3* + pSH62 (2µm *HIS3 GAL1-cre*)<br>*HIP1::TPI1*$_{PRO}$-*HA*$_{3}$-*CAB2-CYC1*$_{TER}$<br>*ENO1::TPI1*$_{PRO}$-*HA*$_{3}$-*CAB3-CYC1*$_{TER}$<br>*MRH1::TPI1*$_{PRO}$-*HA*$_{3}$-*CAB4-CYC1*$_{TER}$<br>*ADH3::TPI1*$_{PRO}$-*HA*$_{3}$-*CAB5-CYC1*$_{TER}$<br>*ZPS1::TPI1*$_{PRO}$-*HA*$_{3}$-*CAB1-CYC1*$_{TER}$
MGY22<br>(derived from MGY18)	*MATα ura3 leu2 trp1 his3* + pSH62 (2µm *HIS3 GAL1-cre*)<br>*HIP1::TPI1*$_{PRO}$-*HA*$_{3}$-*CAB2-CYC1*$_{TER}$<br>*ENO1::TPI1*$_{PRO}$-*HA*$_{3}$-*CAB3-CYC1*$_{TER}$<br>*MRH1::TPI1*$_{PRO}$-*HA*$_{3}$-*CAB4-CYC1*$_{TER}$<br>*ADH3::TPI1*$_{PRO}$-*HA*$_{3}$-*CAB5-CYC1*$_{TER}$<br>*ZPS1::TPI1*$_{PRO}$-*HA*$_{3}$-*CAB1*$_{W331R}$-*CYC1*$_{TER}$<br>*CUP9::TPI1*$_{PRO}$-*HA*$_{3}$-*HAL3*$_{aa\ 260\text{-}495}$-*CYC1*$_{TER}$
MGY24<br>(derived from MGY20)	*MATα ura3 leu2 trp1 his3* + pSH62 (2µm *HIS3 GAL1-cre*)<br>*HIP1::TPI1*$_{PRO}$-*HA*$_{3}$-*CAB2-CYC1*$_{TER}$<br>*ENO1::TPI1*$_{PRO}$-*HA*$_{3}$-*CAB3-CYC1*$_{TER}$<br>*MRH1::TPI1*$_{PRO}$-*HA*$_{3}$-*CAB4-CYC1*$_{TER}$<br>*ADH3::TPI1*$_{PRO}$-*HA*$_{3}$-*CAB5-CYC1*$_{TER}$<br>*ZPS1::TPI1*$_{PRO}$-*HA*$_{3}$-*CAB1-CYC1*$_{TER}$<br>*CUP9::TPI1*$_{PRO}$-*HA*$_{3}$-*HAL3*$_{aa\ 260\text{-}495}$-*CYC1*$_{TER}$
MGY6<br>(derived from BY4741)	*MATa ura3 leu2 his3 met15* + pSH62 (2µm *HIS3 GAL1-cre*)<br>*HIP1::TPI1*$_{PRO}$-*HA*$_{3}$-*CAB2-CYC1*$_{TER}$

MGY13<br>(derived from MGY6)	*MATa ura3 leu2 his3 met15* + pSH62 (2µm *HIS3 GAL1-cre*)<br>*HIP1::TPI1*$_{\mathrm{PRO}}$*-HA*$_{3}$*-CAB2-CYC1*$_{\mathrm{TER}}$<br>*ENO1::TPI1*$_{\mathrm{PRO}}$*-HA*$_{3}$*-CAB3-CYC1*$_{\mathrm{TER}}$
MGY15<br>(derived from MGY13)	*MATa ura3 leu2 his3 met15* + pSH62 (2µm *HIS3 GAL1-cre*)<br>*HIP1::TPI1*$_{\mathrm{PRO}}$*-HA*$_{3}$*-CAB2-CYC1*$_{\mathrm{TER}}$<br>*ENO1::TPI1*$_{\mathrm{PRO}}$*-HA*$_{3}$*-CAB3-CYC1*$_{\mathrm{TER}}$<br>*MRH1::TPI1*$_{\mathrm{PRO}}$*-HA*$_{3}$*-CAB4-CYC1*$_{\mathrm{TER}}$
MGY17<br>(derived from MGY15)	*MATa ura3 leu2 his3 met15* + pSH62 (2µm *HIS3 GAL1-cre*)<br>*HIP1::TPI1*$_{\mathrm{PRO}}$*-HA*$_{3}$*-CAB2-CYC1*$_{\mathrm{TER}}$<br>*ENO1::TPI1*$_{\mathrm{PRO}}$*-HA*$_{3}$*-CAB3-CYC1*$_{\mathrm{TER}}$<br>*MRH1::TPI1*$_{\mathrm{PRO}}$*-HA*$_{3}$*-CAB4-CYC1*$_{\mathrm{TER}}$<br>*ADH3::TPI1*$_{\mathrm{PRO}}$*-HA*$_{3}$*-CAB5-CYC1*$_{\mathrm{TER}}$
MGY19<br>(derived from MGY17)	*MATa ura3 leu2 his3 met15* + pSH62 (2µm *HIS3 GAL1-cre*)<br>*HIP1::TPI1*$_{\mathrm{PRO}}$*-HA*$_{3}$*-CAB2-CYC1*$_{\mathrm{TER}}$<br>*ENO1::TPI1*$_{\mathrm{PRO}}$*-HA*$_{3}$*-CAB3-CYC1*$_{\mathrm{TER}}$<br>*MRH1::TPI1*$_{\mathrm{PRO}}$*-HA*$_{3}$*-CAB4-CYC1*$_{\mathrm{TER}}$<br>*ADH3::TPI1*$_{\mathrm{PRO}}$*-HA*$_{3}$*-CAB5-CYC1*$_{\mathrm{TER}}$<br>*ZPS1::TPI1*$_{\mathrm{PRO}}$*-HA*$_{3}$*-CAB1*$_{\mathrm{W331R}}$*-CYC1*$_{\mathrm{TER}}$
MGY21<br>(derived from MGY17)	*MATa ura3 leu2 his3 met15* + pSH62 (2µm *HIS3 GAL1-cre*)<br>*HIP1::TPI1*$_{\mathrm{PRO}}$*-HA*$_{3}$*-CAB2-CYC1*$_{\mathrm{TER}}$<br>*ENO1::TPI1*$_{\mathrm{PRO}}$*-HA*$_{3}$*-CAB3-CYC1*$_{\mathrm{TER}}$<br>*MRH1::TPI1*$_{\mathrm{PRO}}$*-HA*$_{3}$*-CAB4-CYC1*$_{\mathrm{TER}}$<br>*ADH3::TPI1*$_{\mathrm{PRO}}$*-HA*$_{3}$*-CAB5-CYC1*$_{\mathrm{TER}}$<br>*ZPS1::TPI1*$_{\mathrm{PRO}}$*-HA*$_{3}$*-CAB1-CYC1*$_{\mathrm{TER}}$
MGY23<br>(derived from MGY19)	*MATa ura3 leu2 his3 met15* + pSH62 (2µm *HIS3 GAL1-cre*)<br>*HIP1::TPI1*$_{\mathrm{PRO}}$*-HA*$_{3}$*-CAB2-CYC1*$_{\mathrm{TER}}$<br>*ENO1::TPI1*$_{\mathrm{PRO}}$*-HA*$_{3}$*-CAB3-CYC1*$_{\mathrm{TER}}$<br>*MRH1::TPI1*$_{\mathrm{PRO}}$*-HA*$_{3}$*-CAB4-CYC1*$_{\mathrm{TER}}$<br>*ADH3::TPI1*$_{\mathrm{PRO}}$*-HA*$_{3}$*-CAB5-CYC1*$_{\mathrm{TER}}$<br>*ZPS1::TPI1*$_{\mathrm{PRO}}$*-HA*$_{3}$*-CAB1*$_{\mathrm{W331R}}$*-CYC1*$_{\mathrm{TER}}$<br>*CUP9::TPI1*$_{\mathrm{PRO}}$*-HA*$_{3}$*-HAL3*$_{\mathrm{aa\ 260\text{-}495}}$*-CYC1*$_{\mathrm{TER}}$
MGY25<br>(derived from MGY21)	*MATa ura3 leu2 his3 met15* + pSH62 (2µm *HIS3 GAL1-cre*)<br>*HIP1::TPI1*$_{\mathrm{PRO}}$*-HA*$_{3}$*-CAB2-CYC1*$_{\mathrm{TER}}$<br>*ENO1::TPI1*$_{\mathrm{PRO}}$*-HA*$_{3}$*-CAB3-CYC1*$_{\mathrm{TER}}$<br>*MRH1::TPI1*$_{\mathrm{PRO}}$*-HA*$_{3}$*-CAB4-CYC1*$_{\mathrm{TER}}$<br>*ADH3::TPI1*$_{\mathrm{PRO}}$*-HA*$_{3}$*-CAB5-CYC1*$_{\mathrm{TER}}$<br>*ZPS1::TPI1*$_{\mathrm{PRO}}$*-HA*$_{3}$*-CAB1-CYC1*$_{\mathrm{TER}}$<br>*CUP9::TPI1*$_{\mathrm{PRO}}$*-HA*$_{3}$*-HAL3*$_{\mathrm{aa\ 260\text{-}495}}$*-CYC1*$_{\mathrm{TER}}$
TUY1<br>(derived from JS91.15-23)	*MAT*α *ura3 leu2 trp1 his3* + pSH62 (2µm *HIS3 GAL1-cre*)<br>*ZPS1::TPI1*$_{\mathrm{PRO}}$*-HA*$_{3}$*-CAB1*$_{\mathrm{W331R}}$*-CYC1*$_{\mathrm{TER}}$
TUY4<br>(derived from MGY22)	*MAT*α *ura3 leu2 trp1 his3* + pSH62 (2µm *HIS3 GAL1-cre*)<br>*HIP1::TPI1*$_{\mathrm{PRO}}$*-HA*$_{3}$*-CAB2-CYC1*$_{\mathrm{TER}}$<br>*ENO1::TPI1*$_{\mathrm{PRO}}$*-HA*$_{3}$*-CAB3-CYC1*$_{\mathrm{TER}}$<br>*MRH1::TPI1*$_{\mathrm{PRO}}$*-HA*$_{3}$*-CAB4-CYC1*$_{\mathrm{TER}}$<br>*ADH3::TPI1*$_{\mathrm{PRO}}$*-HA*$_{3}$*-CAB5-CYC1*$_{\mathrm{TER}}$<br>*ZPS1::TPI1*$_{\mathrm{PRO}}$*-HA*$_{3}$*-CAB1*$_{\mathrm{W331R}}$*-CYC1*$_{\mathrm{TER}}$<br>*CUP9::TPI1*$_{\mathrm{PRO}}$*-HA*$_{3}$*-HAL3*$_{\mathrm{aa\ 260\text{-}495}}$*-CYC1*$_{\mathrm{TER}}$<br>*GAT1::TPI1*$_{\mathrm{PRO}}$*-HA*$_{3}$*-FEN2-CYC1*$_{\mathrm{TER}}$
LRY1<br>(derived from MGY22)	*MAT*α *ura3 leu2 trp1 his3* + pSH62 (2µm *HIS3 GAL1-cre*)<br>*HIP1::TPI1*$_{\mathrm{PRO}}$*-HA*$_{3}$*-CAB2-CYC1*$_{\mathrm{TER}}$<br>*ENO1::TPI1*$_{\mathrm{PRO}}$*-HA*$_{3}$*-CAB3-CYC1*$_{\mathrm{TER}}$<br>*MRH1::TPI1*$_{\mathrm{PRO}}$*-HA*$_{3}$*-CAB4-CYC1*$_{\mathrm{TER}}$<br>*ADH3::TPI1*$_{\mathrm{PRO}}$*-HA*$_{3}$*-CAB5-CYC1*$_{\mathrm{TER}}$<br>*ZPS1::TPI1*$_{\mathrm{PRO}}$*-HA*$_{3}$*-CAB1*$_{\mathrm{W331R}}$*-CYC1*$_{\mathrm{TER}}$<br>*CUP9::TPI1*$_{\mathrm{PRO}}$*-HA*$_{3}$*-HAL3*$_{\mathrm{aa\ 260\text{-}495}}$*-CYC1*$_{\mathrm{TER}}$

	pcd1Δ::LEU2
LRY2<br>(derived from TUY4)	*MATα ura3 leu2 trp1 his3* + pSH62 (2µm *HIS3 GAL1-cre*)<br>*HIP1::TPI1*$_{PRO}$*-HA*$_3$*-CAB2-CYC1*$_{TER}$<br>*ENO1::TPI1*$_{PRO}$*-HA*$_3$*-CAB3-CYC1*$_{TER}$<br>*MRH1::TPI1*$_{PRO}$*-HA*$_3$*-CAB4-CYC1*$_{TER}$<br>*ADH3::TPI1*$_{PRO}$*-HA*$_3$*-CAB5-CYC1*$_{TER}$<br>*ZPS1::TPI1*$_{PRO}$*-HA*$_3$*-CAB1*$_{W331R}$*-CYC1*$_{TER}$<br>*CUP9::TPI1*$_{PRO}$*-HA*$_3$*-HAL3*$_{aa\ 260-495}$*-CYC1*$_{TER}$<br>*GAT1::TPI1*$_{PRO}$*-HA*$_3$*-FEN2-CYC1*$_{TER}$<br>*pcd1Δ::LEU2*

Supplementary Table S2: Plasmids used and constructed in this work.

Plasmid	Genetic markers
pRS415	*ARSH4 CEN6 LEU2* (Sikorski and Hieter, 1989)
pRS416	*ARSH4 CEN6 URA3* (Sikorski and Hieter, 1989)
p415-MET25	*ARSH4 CEN6 LEU2 MET25*$_{PRO}$ (Mumberg *et al.*, 1994)
p416-MET25	*ARSH4 CEN6 URA3 MET25*$_{PRO}$ (Mumberg *et al.*, 1994)
p426-MET25HA	2µm *URA3 MET25*$_{PRO}$*-HA*$_3$ (Mumberg *et al.*, 1994)
pSH62	2µm *HIS3 GAL1-cre* (Güldener *et al.*, 1996)
pTM8	*ARSH4 CEN6 URA3 MET25*$_{PRO}$*-CAB1* (wild-type)
pLS12-S8	*ARSH4 CEN6 URA3 MET25*$_{PRO}$*-CAB1*$^{G351S\ W331L}$
pLS12-S9	*ARSH4 CEN6 URA3 MET25*$_{PRO}$*-CAB1*$^{G351S\ D114E}$
pLS12-S10	*ARSH4 CEN6 URA3 MET25*$_{PRO}$*-CAB1*$^{G351S\ F103V}$
pLS12-Y6	*ARSH4 CEN6 URA3 MET25*$_{PRO}$*-CAB1*$^{G351S\ A22G}$
pJO20	*CAB1*
pJO57	*ARSH4 CEN6 URA3 CAB1*
pKH45	*ARSH4 CEN6 LEU2 MET25*$_{PRO}$*-CAB1*
pKH46	*ARSH4 CEN6 LEU2 MET25*$_{PRO}$*-CAB1*^{W331L}
pKH48	*ARSH4 CEN6 LEU2 MET25*$_{PRO}$*-CAB1*$^{Y326A\ F330A}$
pKH49	*ARSH4 CEN6 LEU2 MET25*$_{PRO}$*-CAB1*^{I234E}
pKH50	*ARSH4 CEN6 LEU2 MET25*$_{PRO}$*-CAB1*^{N155V}
pKH51	*ARSH4 CEN6 LEU2 MET25*$_{PRO}$*-CAB1*^{S158V}
pKH52	*ARSH4 CEN6 LEU2 MET25*$_{PRO}$*-CAB1*^{R173A}
pKH53	*ARSH4 CEN6 LEU2 MET25*$_{PRO}$*-CAB1*^{A233E}
pKH54	*ARSH4 CEN6 LEU2 MET25*$_{PRO}$*-CAB1*^{W331R}
pSBS5	2µm *URA3 MET25*$_{PRO}$*-HA*$_3$*-CAB1* (Olzhausen *et al.*, 2009)
pJO3	2µm *URA3 MET25*$_{PRO}$*-HA*$_3$*-CAB3*
pJO73	2µm *URA3 MET25*$_{PRO}$*-HA*$_3$*-PANK3* (Olzhausen *et al.*, 2009)
pEB5	2µm *URA3 MET25*$_{PRO}$*-HA*$_3$*-CAB1*$^{Y326A\ F330A}$
pEB6	2µm *URA3 MET25*$_{PRO}$*-HA*$_3$*-cab1*^{I234E}
pEB8	2µm *URA3 MET25*$_{PRO}$*-HA*$_3$*-CAB1*^{W331L}
pEB22	2µm *URA3 MET25*$_{PRO}$*-HA*$_3$*-CAB1*^{N155V}
pEB23	2µm *URA3 MET25*$_{PRO}$*-HA*$_3$*-cab1*^{S158V}
pEB25	2µm *URA3 MET25*$_{PRO}$*-HA*$_3$*-cab1*^{R173A}
pEB26	2µm *URA3 MET25*$_{PRO}$*-HA*$_3$*-CAB1*^{A233E}
pEB27	2µm *URA3 MET25*$_{PRO}$*-HA*$_3$*-CAB1*^{W331R}
pGE7	*ARSH4 CEN6 URA3 CAB4*
pGE8	*ARSH4 CEN6 LEU2 CAB4*
pGE9	*ARSH4 CEN6 URA3 CAB5*
pGE10	*ARSH4 CEN6 LEU2 CAB5*
pLS14	2µm *URA3 MET25*$_{PRO}$*-HA*$_3$*-hCOASY* (full-length)

pLS15	2µm *URA3 MET25*$_{PRO}$-*HA*$_3$-*hCOASY* (aa 30-564)
pLS20	*ARSH4 CEN6 LEU2 MET25*$_{PRO}$-*hCOASY* (full-length)
pSBS7	*cab1*Δ*::HIS3* (Olzhausen *et al*., 2009)
pSB2	*cab4*Δ*::HIS3* (Olzhausen *et al*., 2009)
pSB5	*cab5*Δ*::HIS3* (Olzhausen *et al*., 2009)
pLR2	*pcd1*Δ*::LEU2*
pLEUTEX3	*TPI1*$_{PRO}$-HA$_3$-*CYC1*$_{TER}$::loxP::*LEU2*::loxP
pLEUTEX-CAB1	*ZPS1*-5´-flank::*TPI1*$_{PRO}$-HA$_3$-*CAB1-CYC1*$_{TER}$::*ZPS1*-3´-flank
pLEUTEX-CAB1 W331R	*ZPS1*-5´-flank::*TPI1*$_{PRO}$-HA$_3$-*CAB1*$_{W331R}$-*CYC1*$_{TER}$::*ZPS1*-3´-flank
pLEUTEX-CAB2	*HIP1*-5´-flank::*TPI1*$_{PRO}$-HA$_3$-*CAB2-CYC1*$_{TER}$::*HIP1*-3´-flank
pLEUTEX-CAB3	*ENO1*-5´-flank::*TPI1*$_{PRO}$-HA$_3$-*CAB3-CYC1*$_{TER}$::*ENO1*-3´-flank
pLEUTEX-CAB4	*MRH1*-5´-flank::*TPI1*$_{PRO}$-HA$_3$-*CAB4-CYC1*$_{TER}$::*MRH1*-3´-flank
pLEUTEX-CAB5	*ADH3*-5´-flank::*TPI1*$_{PRO}$-HA$_3$-*CAB5-CYC1*$_{TER}$::*ADH3*-3´-flank
pLEUTEX-HAL3	*CUP9*-5´-flank::*TPI1*$_{PRO}$-HA$_3$-*HAL3*$_{aa260-495}$-*CYC1*$_{TER}$::*CUP9*-3´-flank
pLEUTEX-FEN2	*GAT1*-5´-flank::*TPI1*$_{PRO}$-HA$_3$-*FEN2-CYC1*$_{TER}$::*GAT1*-3´-flank

aa, amino acids; PRO, promoter; TER, terminator.

Supplementary Table S3: Oligonucleotides used in this work.

**Name**	**Gene**	**Position**	**Sequence (5´-3´)**
**ORF-Primer**			
CAB1-Bam-Start	*CAB1*	+1/+19	gactggatccATGCCGCGAATTACTCAAG
CAB1-Hind-Stop	*CAB1*	+1104/+1084	gactaagcttCTACGTACTTGTTTTCTTAGT
YIL083C-BamHI	*CAB2*	+1/+20	gactggatccATGCCACCTCTACCCGTGCT
YIL083C-XhoI	*CAB2*	+1098/+1075	gactctcgagTTATTTCGTAGCTAGCTTAGTTTT
YKL088W-Start	*CAB3*	+1/+20	gactggatccATGACGGATGAAAAAGTGAA
YKL088W-Stop	*CAB3*	+1716/+1693	gactaagcTTAAACTTGCGTTTTCACGTCTTC
CAB4-Bam-Start	*CAB4*	+1/+20	gactggatccATGGTTGAGGAAAATTCCAG
CAB4-Hind-Stop	*CAB4*	+918/+897	gactaagcTTATACGCAAGGGTTTTGTGGA
CAB5-Bam-Start	*CAB5*	+1/+20	gactggatccATGCTGGTAGTGGGATTGAC
CAB5-Cla-Stop	*CAB5*	+726/+705	gactatcgatTTATACCGCTGAAGACTTTTTA
Hal3-BglII Start260	*HAL3*	+778/+798	gatcagatctAAAATGGATGATGGAAAATTGCACGTG
Hal3-HindIII Stop495	*HAL3*	+1485/+1464	gatcaagcttTTAGTTATTTTTTGGGTACCCACCC
FEN2-BamHI Start	*FEN2*	+1/+22	gatcggatccATGATGAAGGAATCGAAATCTA
FEN2-HindIII Stop	*FEN2*	+1539/+1517	gatcaagcttCTATCGGAAAGGATTTGAAATTT
**Mutagenic primer**			
CAB1-	*CAB1*	+445/+483	TACCCATACCTTCTAGTC**GTG**ATAGGGT-

N155V-F			CGGGTGTCTCA
CAB1-N155V-R	*CAB1*	+483/+445	TGAGACACCCGACCCTAT**CAC**GACTAGA-AGGTATGGGTA
CAB1-S158V-F	*CAB1*	+453/+489	CCTTCTAGTCAATATAGGG**GTG**GGTGTC-TCAATATTA
CAB1-S158V-R	*CAB1*	+489/+453	TAATATTGAGACACC**CAC**CCCTATATTGA-CTAGAAGG
CAB1-R173A-F	*CAB1*	+499/+537	GAACCAAACAATTTTAGT**GCC**GTAGGCG-GTTCTTCACTG
CAB1-R173A-R	*CAB1*	+537/+499	CAGTGAAGAACCGCCTAC**GGC**ACTAAAA-TTGTTTGGTTC
CAB1-I234E-F	*CAB1*	+682/+717	GGTCTAAAGTCGTCAGCT**GAG**GCAAGTT-CATTTGGT
CAB1-I234E-R	*CAB1*	+717/+682	ACCAAATGAACTTGC**CTC**AGCTGACGAC-TTTAGACC
CAB1-A233E-F	*CAB1*	+682/+716	GGTCTAAAGTCGTCA**GAG**ATTGCAAGTT-CATTTGG
CAB1-A233E-R	*CAB1*	+716/682	CCAAATGAACTTGCAAT**CTC**TGACGAC-TTTAGACC
CAB1-W331R-F	*CAB1*	+972/1013	GAGCTACGCTATTAATTTT**CGG**TCACAA-GGATCAAAGCAAGC
CAB1-W331R-R	*CAB1*	+1013/972	CGTTGCTTTGATCCTTGTGA**CCG**AAAATT-AATAGCGTAGCTC
CAB1-W331L-F	*CAB1*	+972/+1013	GAGCTACGCTATTAATTTT**TTG**TCACAA-GGATCAAAGCAAGC
CAB1-W331L-R	*CAB1*	+1013/+972	CGTTGCTTTGATCCTTGTGA**CAA**AAAATT-AATAGCGTAGCTC
CAB1-Y326A F330A-F	*CAB1*	+958/+1008	ACCATGAACACTTTGAGC**GCG**GCTATTAAT-**GCG**TGGTCACAAGGATCAAAG
CAB1-Y326A F330A-R	*CAB1*	+1008/+958	CTTTGATCCTTGTGACCA**CGC**AATAATAGC-**CGC**GCTCAAAGTGTTCATGGT
**Primers for amplification of flanking regions to be inserted into pLEUTEX cassettes**			
ADH3 5F SacI	*ADH3*	-2100/-2081	gatcgagctcCTGCATGTTTACAAGGCAAA
ADH3 5F BglII	*ADH3*	-1631/-1650	gatcagatctTGGTCAAGACCAGAATTACT
ADH3 3F NheI	*ADH3*	-1550/-1531	gatcgctagcCAAGGAGTAGCTTCCTTCAA
ADH3 3F KpnI	*ADH3*	-1131/-1150	gatcggtaccCATTGTGCAAAGACCCATGC
ADH3 Veri	*ADH3*	-2140/-2121	CAGAAGCTTTCGTGAGATAC
CUP9 5F SacI	*CUP9*	-1570/-1551	gatcgagctcGACGGCTTGTAACGGGTGCC
CUP9 5F BglII	*CUP9*	-1141/-1160	gatcagatctAGGGGCCCGACAATATTGCT
CUP9 3F NheI	*CUP9*	-1030/-1011	gatcgctagcGTAGTATGCCTCCGAAGCTC
CUP9 3F KpnI	*CUP9*	-601/-620	atcggtaccGTGCTCAGAAAACCCCGTGG
CUP9 Veri	*CUP9*	-1620/-1601	GATATTTGAATCAGCTGGAG
ENO1 5F SacI	*ENO1*	-1580/-1561	gatcgagctcTACATTCATGTTTGCACGCT
ENO1 5F BglII	*ENO1*	-1231/-1250	gatcagatctTCTGGATAAGTGCTGTGCAT
ENO1 3F	*ENO1*	-1106/-1086	gatcgctagcAATGGCCCTATATTGAAAGC

NheI			
ENO1 3F KpnI	*ENO1*	-771/-790	gatcggtaccGCTCAAGTGCCCGCGGCATC
ENO1 Veri	*ENO1*	-1210/-1191	TGACTCAAATTCTTCTCGAC
GAT1 5F SacI	*GAT1*	+1881/+1900	gatcgagctcAACCGAGACCTGAACATCCT
GAT1 5F BamHI	*GAT1*	+2320/+2301	gatcggatccAGCGGGAAACTCCTTAGTGT
GAT1 3F NheI	*GAT1*	+2451/+2460	gatcgctagcGCCCCAAATGTCAGTGATGG
GAT 3F KpnI	*GAT1*	+2870/+2851	gatcggtaccAGAAACGATATTAAGCCTGG
GAT1 Veri	*GAT1*	+1811/+1830	CTAAACCAGTTATCCTAGGC
HIP1 5F SacI	*HIP1*	-1573/-1554	gactgagctcTTGTCTGCCGAAATTCTGTG
HIP1 5F BamHI	*HIP1*	-1162/-1171	gactggatccAGTAGCTTGTGAACGATAGG
HIP1 3F NheI	*HIP1*	-960/-941	gactgctagcTTTACCCCTCTCCACAGATC
HIP1 3F KpnI	*HIP1*	-636/-655	gactggtaccCCAGGTTGAATACAAAACGG
HIP1 5-Veri	*HIP1*	-1620/-1601	ATTGAGCCACCGCTTCATCT
MRH1 5F SacI	*MRH1*	-1860/-1841	gatcgagctcTCTTGTTCCCATCTACGGGT
MRH1 5F BglII	*MRH1*	-1471/-1490	gatcagatctGGAAGCGTTGGAAAAGAAGG
MRH1 3F NheI	*MRH1*	-1230/-1211	gatcgctagcCTCCTCGAGACAAGGTCGCT
MRH1 3F KpnI	*MRH1*	-851/-870	gatcggtaccGTTTGGGTCTCTTGGTAACC
MRH1 Veri	*MRH1*	-1940/-1921	ACTTGGCGCGCCGACATTTG
ZPS1 5F SacI	*ZPS1*	-2380/-2361	atcgagctcAGGCGCGTGTCTACCTCGTA
ZPS1 5F BglII	*ZPS1*	-1931/-1150	gatcagatctGGCCTCGGCAACTTTCAGGG
ZPS1 3F NheI	*ZPS1*	-1560/-1541	gatcgctagcGGTATGATCTACCCCATTGC
ZPS1 3F KpnI	*ZPS1*	-1241/-1250	gatcggtaccCCGCGAAGACTACGCAAATA
ZPS1 Veri	*ZPS1*	-2430/-2411	AGTTAGTGGTTGCCTTCCGT
**Construction of pLEUTEX3**			
LEU2-LBN			gactggatccatgcatCAACCCTTAATATAACTTCGTATA
LEU2-RXN			gactctcgaggctagcACCTAATAACTTCGTATAGCAT
MET25HAX			gacttctagaAAAATGTATCCTTATGATGTTCC
CYC1TER NSI			gactatgcatCGGCCGCAAATTAAAGCCTT
TPI1-Prom	*TPI1*	-600/-580	gactgagctcagatctGAGACCTAACTACATAGTGT
TPI1PR-XB	*TPI1*	-26/-5	gacttctagaGTTTATGTATGTGTTTTTTGT

Authentic gene sequences are shown in capital letters; auxiliary sequences are depicted in lower case letters. Bold letters indicate the sequence of mutations introduced.

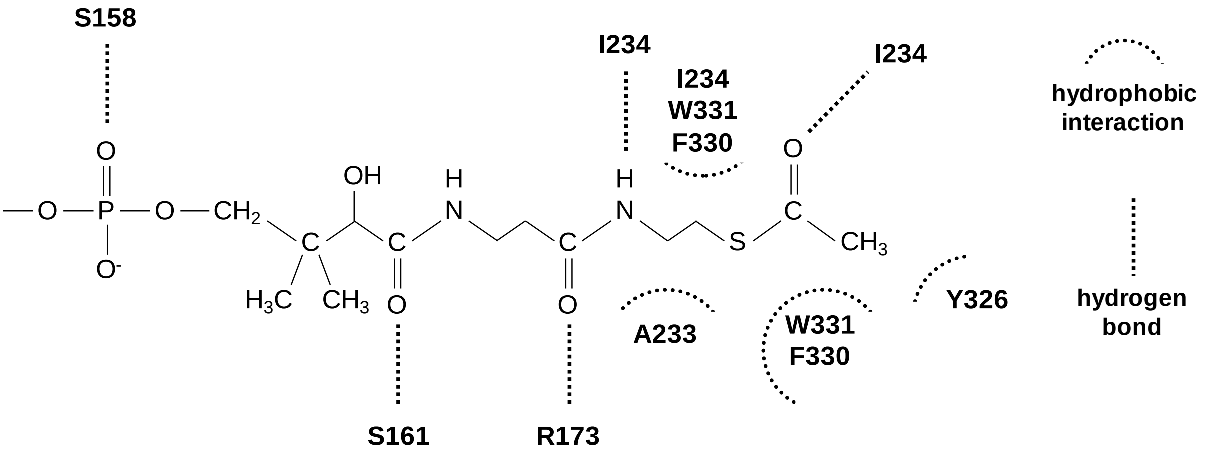

Supplementary Figure S1: Presumed binding of acetyl-CoA to the substrate binding site of yeast PanK (derived from the crystal structure of human PanK3 isoenzyme in the presence of acetyl-CoA; taken from Hong *et al*. [2007] and assigned to the yeast enzyme)

```
Cab1 (Sc)  MPRITQEISYNCDYGDNTFNLAIDIGGTLAKVVFSPIHSNRLMFYTIETEKIDKFMELLH  60
CoaA (Sa)  ------------------MKVGIDAGGTLIKIVQE--QDNQRTFKTELTKNIDQVVEWLN  40

Cab1 (Sc)  SIIKEHNNGCYRMTHIIATGGGAFKFYDLLYENFPQIKGISRFEEMEGLIHGLDFFIHEI 120
CoaA (Sa)  QQQ---------IEKLCLTGGNAGVIAENINIP------AQIFVEFDAASQGLGILLKEQ  85

Cab1 (Sc)  PDEVFTYNDQDGERIIPTSSGTMDSKAIYPYLLVNIGSGVSILKVTEPNNFSRVGGSSLG 180
CoaA (Sa)  G------------------------HDLADYIFANVGTGTSLHYFDGQ-SQRRVGGIGTG 120

Cab1 (Sc)  GGTLWGLLSLITGAQTYDQMLDWAQEGDNSSVDMLVGDIYGTDYNKIGLKSSAIASSFGK 240
CoaA (Sa)  GGMIQGLGYLLSQITDYKQLTDMAQHGDRNTIDLKVRHIY-KDTE-PPIPGDLTAANFGH 178

Cab1 (Sc)  VFQNRMTSNKSLENNENKLYSSHESIEKNNGQMFKNPDICKSLLFAISNNIGQIAYLQAK 300
CoaA (Sa)  VLHHLDADFTPSN-------------------------KLAAVIGVVGEVVTTMAITVAR 213

Cab1 (Sc)  INNIQNIYFGGSYTRGHLTTMNTLSYAINFWSQGSKQAFFLKHEGYLGAMGAFLSASRHS 360
CoaA (Sa)  EFKTENIVYIGSSFHNNALLRKVVEDYTVLRG---CKPYYVENGAFSGAIGALYLEK*   267

Cab1 (Sc)  STKKTST* 367
```

Supplementary Figure S2: Similarity of pantothenate kinases from *Saccharomyces cerevisiae* (Sc; *CAB1* gene product) and *Staphylococcus aureus* (Sa; *coaA* gene product). Conserved amino acids are shaded in grey. Amino acids used for mutational analysis of Cab1 are indicated in bold and by inverse shading.

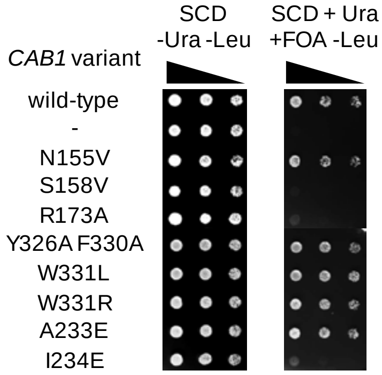

Supplementary Figure S3: Functional analysis of *CAB1* variants by plasmid shuffling. Strain JS19.1 with a chromosomal *cab1*Δ null allele and *ARS CEN URA3 CAB1* rescue plasmid (pJO57) was transformed with *ARS CEN LEU2* plasmids containing the *CAB1* variants indicated. All transformants were able to grow on double-selective synthetic medium (SCD-Ura-Leu) while only functional variants of *CAB1* allowed growth on FOA-containing medium after loss of the rescue plasmid. FOA: 5-Fluoroorotic acid.

Supplementary description: Construction of pLEUTEX3

Plasmid pLEUTEX3 was constructed in a three-step-procedure:

1. Insertion of selection marker *LEU2* into pBluescript

Plasmid pJS491 (unpublished) had been constructed by inserting the selection marker *LEU2* flanked by loxP sites into the backbone of pUC19. Using pJS491 as a template together with PCR primers LEU2-LBN (introduces restriction sites for *Bam*HI and *Nsi*I) and LEU2-RXN (introduces restriction sites for *Xho*I and *Nhe*I), a 1.73 kb loxP::*LEU2*::loxP fragment was amplified, digested with *Bam*HI + *Xho*I and inserted into *Bam*HI + *Xho*I cleaved pBluescript II KS(+) to give pLEUTEX1.

2. Insertion of the HA_3 epitope and *CYC1* terminator

Using p425-MET25HA as a template together with PCR primers MET25HAX (introduces restriction site for *Xba*I) and CYC1TERNSI (introduces restriction site for *Nsi*I), a 0.4 kb fragment was amplified. This PCR fragment was digested with *Xba*I + *Nsi*I and inserted into pLEUTEX1 which had been also digested with these restriction enzymes (giving pLEUTEX2).

3. Insertion of *TPI1* promoter

The *TPI1* promoter was amplified using chromosomal DNA as a template together with PCR primers TPI1-Prom (introduces restriction sites for *Sac*I + *Bgl*II) and TPI1PR-XB (introduces restriction site for *Xba*I). The resulting 0.6 kb fragment was digested with *Sac*I + *Nsi*I and inserted into pLEUTEX2 which had been digested with the same restriction enzymes (to give pLEUTEX3).

Using pLEUTEX3, gene-specific expression cassettes can be constructed by inserting the coding region of a CoA biosynthetic gene (*TPI1-CAB* fusion) and ligating flanking sequences (about 400 bp; left flank: *Sac*I + *Bgl*II fragment, right flank: *Nhe*I + *Kpn*I fragment) on both sides. Following release of the expression cassette by cleavage with *Sac*I + *Kpn*I, recombination can occur at the chromosomal site homologous to the flanking region.

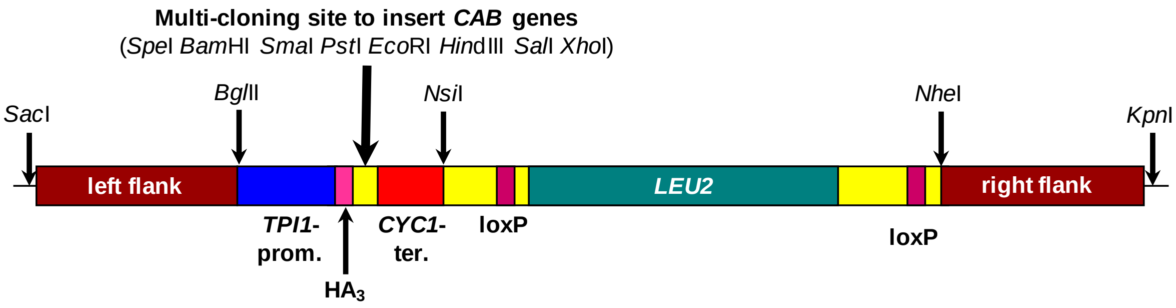

Supplementary Figure S4: Functional elements of pLEUTEX expression cassettes suitable for repeated integration of CoA biosynthetic genes. Selection marker *LEU2* flanked by loxP sites can be removed after a successful transformation by induction of cre recombinase which targets loxP sites. Several unique restriction sites following the *TPI1*-HA promoter/epitope segment allow insertion of *CAB* reading frame cassettes. For integration of the expression/selection cassette by homologous recombination, flanking sequences from a genomic site of choice must be inserted by using restriction sites *Sac*I + *Bgl*II (left flank) and *Nhe*I + *Kpn*I (right flank), respectively. Prior to yeast transformation, expression cassettes together with flanking regions can be released by cleavage with *Sac*I + *Kpn*I.

Supplementary Method: Enzymatic assay of CoA + acetyl-CoA concentrations

The cycling assay described by Bergmeyer (1974) allows repeated use of CoA and acetyl-CoA by regeneration of CoA to acetyl-CoA catalyzed by phosphotransacety-lase (PAT), leading to signal amplification. Using the citrate synthase (CS) reaction, acetyl-CoA and oxaloacetate react to give citrate. Oxaloacetate is synthesized from malate by NAD-dependent malate dehydrogenase which serves as a preceding indicator reaction, leading to an increase of absorbance by NADH at 340 nm.

NEM (N-ethylmaleimide) can react with free SH group of CoA so that only acetyl-CoA is detected.

Various samples containing CoA + acetyl-CoA with known concentrations (ratio 1:1) were used for calibration and validation of CoA assays in protein-free metabolite extracts. Dilution of these extracts as a result of protein removal was considered by calculation of the dilution factor.

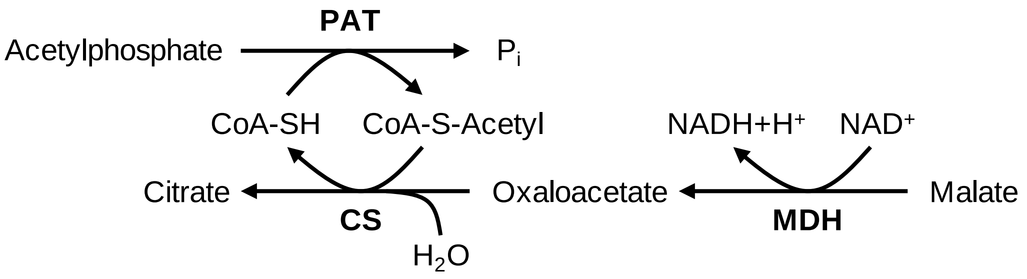

Reaction mixture (final concentrations):

- 140 mM Triethanolamine/HCl
- 10.5 mM Malate (potassium salt)
- 0.46 mM DTT or 0.46 mM NEM
- 1.4 mM NAD
- 4.6 mM Acetylphosphate (lithium salt)
- 7000 U/l PAT
- 1300 U/l CS
- 9800 U/l MDH
